# Thicker eggshells are not predicted by host egg ejection behaviour in four species of Australian cuckoo

**DOI:** 10.1038/s41598-022-09872-9

**Published:** 2022-04-15

**Authors:** Clare E. Holleley, Alice C. Grieve, Alicia Grealy, Iliana Medina, Naomi E. Langmore

**Affiliations:** 1 Australian National Wildlife Collection, National Research Collections Australia, CSIRO, Canberra, ACT 2601 Australia; 2 Langmore Group, Research School of Biology, Building 46, Australian National University, Canberra, ACT 0200 Australia; 3 School of BioSciences, University of Melbourne, Royal Parade, VIC 3010 Australia

**Keywords:** Coevolution, Evolutionary ecology

## Abstract

Defences of hosts against brood parasitic cuckoos include detection and ejection of cuckoo eggs from the nest. Ejection behaviour often involves puncturing the cuckoo egg, which is predicted to drive the evolution of thicker eggshells in cuckoos that parasitise such hosts. Here we test this prediction in four Australian cuckoo species and their hosts, using Hall-effect magnetic-inference to directly estimate eggshell thickness in parasitised clutches. In Australia, hosts that build cup-shaped nests are generally adept at ejecting cuckoo eggs, whereas hosts that build dome-shaped nests mostly accept foreign eggs. We analysed two datasets: a small sample of hosts with known egg ejection rates and a broader sample of hosts where egg ejection behaviour was inferred based on nest type (dome or cup). Contrary to predictions, cuckoos that exploit dome-nesting hosts (acceptor hosts) had significantly thicker eggshells relative to their hosts than cuckoos that exploit cup-nesting hosts (ejector hosts). No difference in eggshell thicknesses was observed in the smaller sample of hosts with known egg ejection rates, probably due to lack of power. Overall cuckoo eggshell thickness did not deviate from the expected avian relationship between eggshell thickness and egg length estimated from 74 bird species. Our results do not support the hypothesis that thicker eggshells have evolved in response to host ejection behaviour in Australian cuckoos, but are consistent with the hypothesis that thicker eggshells have evolved to reduce the risk of breakage when eggs are dropped into dome nests.

## Introduction

Avian obligate brood parasites, such as cuckoos, minimise their reproductive costs by laying their eggs in the nests of other birds to be raised by the host. As resources are diverted away from the host’s own young, pressure is placed on hosts to evade parasitism, which in turn places pressure on parasites to evolve ever more elaborate tactics to evade detection by hosts. This results in a ‘co-evolutionary arms race’^1^.

Brood parasitism imposes heavy costs on hosts. Cuckoo nestlings usually evict or outcompete host nestlings, so hosts typically fail to fledge any of their own young, and thus selection favours the evolution of host defences. Host defences include mobbing of adult cuckoos^2^, and rejection of cuckoo eggs^3,4^ or chicks^5–7^. Host defences have, in turn, selected for a suite of adaptations in brood parasites that facilitate parasitism of host nests, including rapid egg laying^8^, and mimicry of host eggs^3^ or chicks^9,10^. Specifically, some brood parasites have evolved mimicry of host egg shape and colour^11,12^, chick begging calls^13,14^ and chick morphology (e.g. skin colour, mouth and gape patterns^9,10^, as well as adult mimicry of non-parasitic birds^15–20^. An alternative strategy employed by other brood parasites is to lay dark eggs that are cryptic rather than mimetic when the nest is dome-shaped, causing the egg to blend into the dimly lit interior of the nest, thereby escaping detection by the host^15,21,22^. The interactions between brood parasites and their hosts provide some of the most well-studied examples of co-evolutionary arms races in nature^1^.

Although previous studies have demonstrated cases where traits expressed by brood parasites (such as egg colour, size and pattern mimicry) have arisen due to co-evolution with the host, it is unclear whether other phenotypes are the outcome of co-evolution or other processes. For example, the eggshells of many cuckoo species are thicker and stronger than those of their hosts, relative to body size^23–26^, but there is conflicting evidence about whether eggshell thickness has evolved through co-evolution with hosts. Eggshell thickness is a physiologically constrained trait. The eggshell must be simultaneously thick enough to avoid breakage and mediate UV exposure during embryonic development, and yet sufficiently thin for efficient embryonic gas exchange and to allow the chick to hatch^27–30^. Thus, it is an interesting case study of how co-evolutionary pressures operate in the presence of tight physiological constraints.

The evolution of thicker eggshells is predicted to be an effective counter strategy to host defence behaviour because the cuckoo eggs are more difficult for the host to puncture, which impedes the host’s ability to remove the egg. This is called the ‘puncture resistance hypothesis’^24,31^. There are also additional theories that predict the evolution of thicker eggshells in cuckoos that are unrelated to host egg ejection behaviour. For example, the ‘impact resistance hypothesis’^23^ predicts that cuckoo chick survival is increased by reducing the damage sustained during rapid egg laying events or when the egg is laid from a height above the nest^32^. The ‘heat retention hypothesis’^33^ predicts that cuckoo chick survival is increased by accelerated developmental rates allowing early hatching and eviction of the host’s eggs^34,35^. Finally, the ‘multiple parasitism’ hypothesis predicts that a thicker eggshell protects cuckoo eggs from being damaged by other cuckoos in repeatedly parasitised nests^26^. Thus, there is a general expectation that selection favours the evolution of thicker eggshells in brood parasites.

The experimental evidence regarding the evolution in brood parasites is somewhat conflicting. An early study compared the eggshell thickness of *Cuculus/Cacomantis/Chalcites/Chrysococcyx* genera of cuckoos with those of *Clamator* cuckoos and found that the eggshell thickness of the former group did not differ significantly from those of their hosts, whereas the latter group had significantly thicker eggshells than their hosts^26^. However, a more in-depth study of *Cuculus canorus* and its hosts revealed that races that exploit hosts with strong egg ejection behaviour have thicker shells relative to their hosts than races that exploit less discriminating species^36^. This suggests that cuckoos within *Cuculus/Cacomantis/Chalcites/Chrysococcyx* cuckoos included in the Brooker & Brooker (1991)^26^ study may differ in their eggshell thickness according to the ejection behaviour of their hosts.

Here we investigate whether co-evolutionary interactions with hosts drive the evolution of eggshell thickness in brood parasites, by studying a range of host species that vary in their egg ejection behaviour. We predict that if thicker eggshells evolve to reduce the risk of egg ejection by hosts, cuckoo eggshells should be thicker than those of their hosts only in cuckoo species that exploit egg-ejecting hosts. Conversely, if thicker eggshells have evolved to reduce the risk of breakage, eggshell thickness—while still being thicker relative to their host—should not differ between cuckoos that exploit egg-ejecting versus egg accepting hosts.

## Results

There was a high degree of repeatability between independent thickness measurements in 10 eggs that were measured multiple times. The mean apex average measure intra-class correlation coefficient (ICC) was 0.961 with a 95% confidence interval from 0.912 to 0.989 (*F*_9,90_ = 25.6, *P* < 0.0001). The mean meridian average measure ICC was 0.961 with a 95% confidence interval from 0.913 to 0.989 (*F*_9,90_ = 25.9, *P* < 0.0001) (Supplementary Information Sect. 4.0).

**Figure 1:**
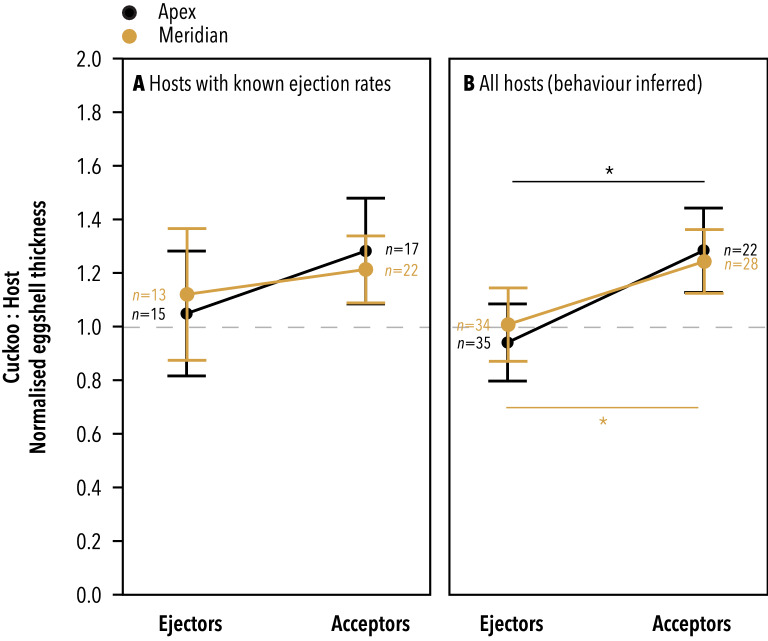
The ratio of normalised eggshell thickness between cuckoos and their ejector versus acceptor hosts. (**A**) In the smaller sample size dataset that includes only hosts with known egg ejection rates, there is no significant difference in the cuckoo:host ratio of eggshell thickness between acceptors and ejectors at either point measured (apex: *F*_1,1.98_ = 2.31, *P* = 0.27; meridian: *F*_1,5.6_ = 0.99, *P* = 0.36). (**B**) When all hosts are considered and ejection behaviour is inferred from nest type, cuckoos that exploit acceptor hosts have thicker eggshells relative to their hosts than cuckoos that exploit ejectors, at both the apex (*F*_1,12.47_ = 7.72, *P* = 0.02) and the meridian of the egg (*F*_1,14.32_ = 8.86_,_ *P* = 0.01).

**Figure 2:**
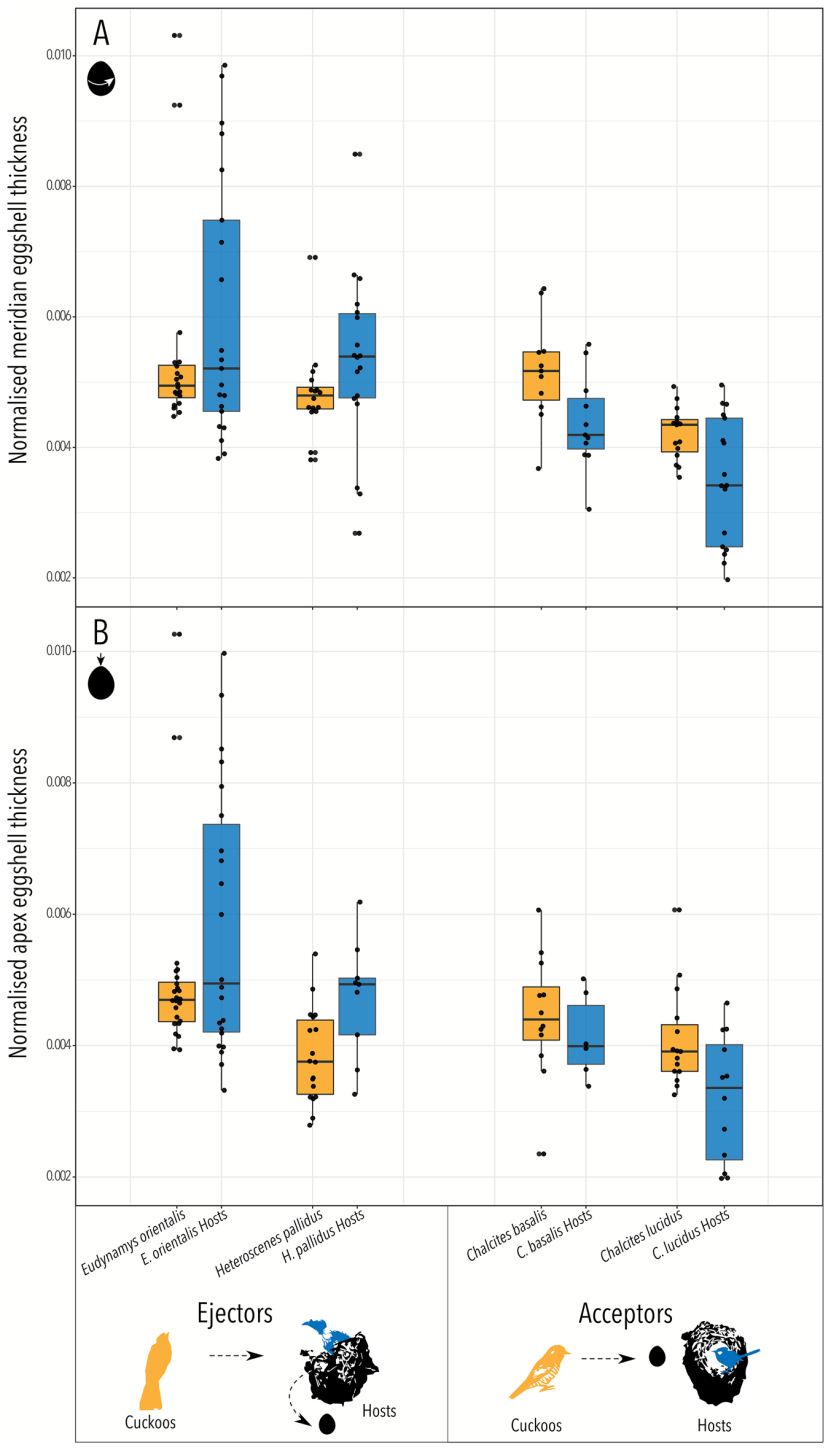
Eggshell thickness of cuckoos (orange) and their hosts (blue). Eggshell thickness was measured at two points on the egg: (**A**) the meridian of the egg, which is the circumference around the widest part of the egg and (**B**) the apex of the egg, which is the most conical end opposite the air sac. Eggshell thickness was normalised for interspecies comparisons by dividing the mean eggshell thickness of each egg by its length. The total eggshell thickness distribution is shown as black dots (Supplementary data file 1). Box plots are the median, interquartile range (Q1–Q3) and range (min–max). Raw data prior to normalisation are displayed in Fig. S3.

Overall, the thickness of cuckoo eggshells relative to their length was not significantly different from that of their hosts (Wilcoxon Signed-Rank Test, Apex: *S* = 91*, N* = 47*, P* = 0.34; Meridian: *S* = -114*, N* = 59*, P* = 0.39). Moreover, for hosts with known egg ejection rates, there was no significant difference in the relative thickness of cuckoo:host eggshells between cuckoos that exploit ejector hosts and those that exploit acceptors (Figs. 1 and 2) at the apex of the egg (*F*_1,1.98_ = 2.31, *P* = 0.27) or at the meridian of the egg (*F*_1,5.6_ = 0.99, *P* = 0.36). However, when considering hosts with both known and unknown egg ejection rates, cuckoos that exploit dome-nesting acceptors showed a significant tendency to have thicker eggshells relative to their hosts than cuckoos that exploit cup-nesting ejectors, at both the apex (*F*_1,12.47_ = 7.72, *P* = 0.02) and the meridian of the egg (*F*_1,14.32_ = 8.86_,_ *P* = 0.01) (Figs. 1 and 2).

**Figure 3:**
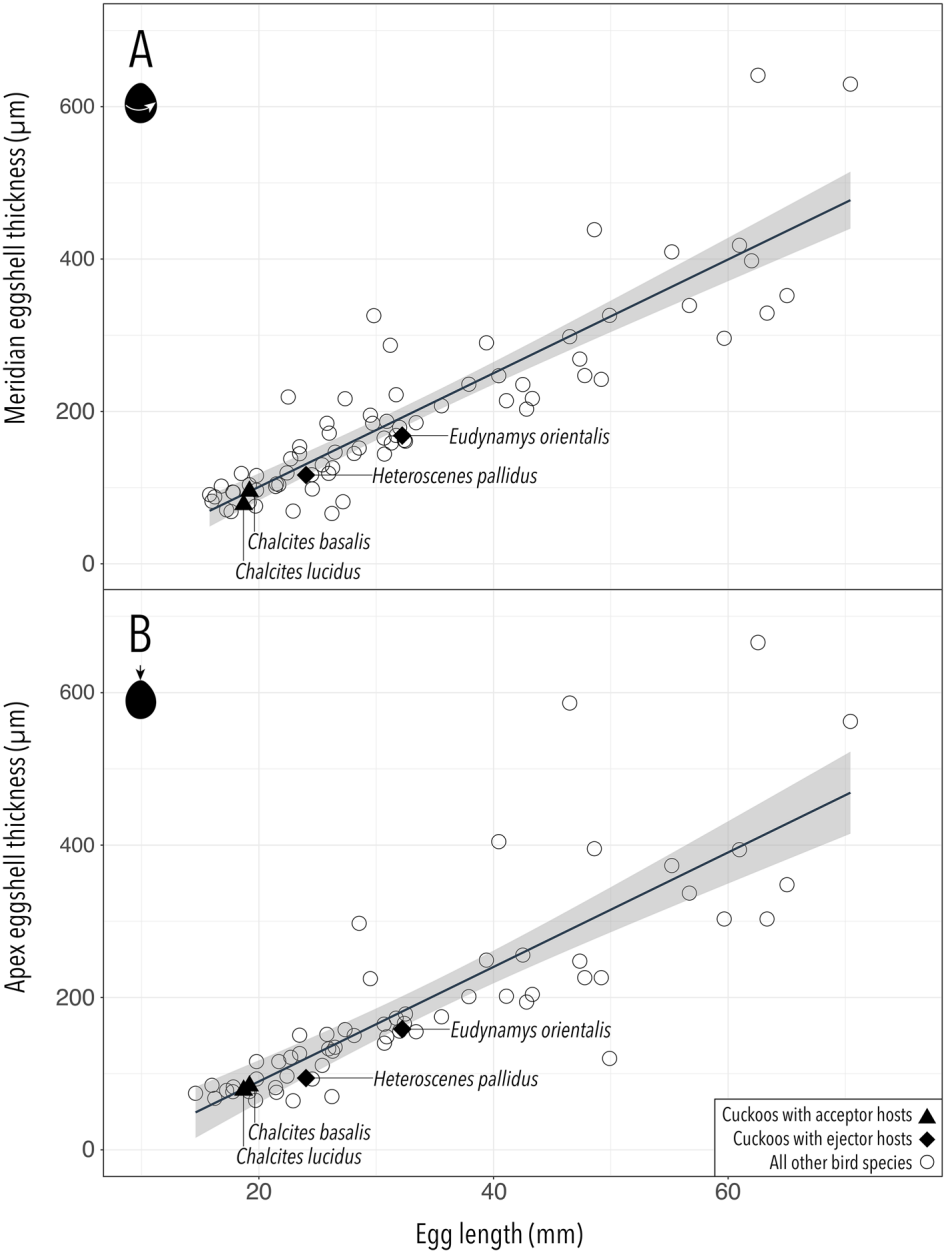
Eggshell thickness as a function of egg length in avian species. Eggshell thickness was measured at two points on the egg: (**A**) the meridian of the egg, which is the circumference around the widest part of the egg (*N* = 73) and (**B**) the apex of the egg, which is the most conical end opposite the air sac (*N* = 59). Linear regression was conducted on species that do not employ a brood parasitic reproductive strategy (circles). Brood parasitic cuckoos were plotted separately (filled black markers). Cuckoo species that parasitise acceptor hosts without egg ejection (black triangles) and cuckoo species that exploit egg-ejecting hosts (black diamonds) fell within the 95% confidence limit (grey shaded area) predicted for non-parasitic species. Figures of residuals when using phylogenetic correction are presented in supplementary material (Figs. S5 and S6).

As expected, there was a significant positive relationship between egg length and eggshell thickness in the 74 avian species examined (Fig. 3). The results of the phylogenetically corrected and uncorrected linear regression analyses are qualitatively identical and the strength of the association between eggshell size and thickness is comparable. Before correcting for phylogenetic relatedness, egg length predicted meridian eggshell thickness slightly better than apex eggshell thickness (Linear regression meridian: *y* = 7.4636*x*−48.406; *R*^2^ = 0.7822; *P* < 0.0001. Linear regression apex: *y* = 7.5163*x*−60.578; *R*^2^ = 0.6768; *P* < 0.0001). There was a slightly higher sample size in the meridian data set (*N* = 73 avian species + 4 cuckoos) compared to the apex data set (*N* = 59 avian species + 4 cuckoos). Sample sizes differed between apex and meridian estimates because some measurements were not possible due to eggshell damage or blow hole placement. The four species of brood parasitic cuckoos adhered to the general avian relationship between egg length and eggshell thickness, occurring within the 95% confidence interval for all other species (Fig. 3). The residuals (phylogenetic and non-phylogenetic) were not distinct from those of other species, and cuckoos did not have particularly thick eggshells compared to other species (Figs. S5 and S6).

## Discussion

We set out to determine if eggshell thickness in Australian avian brood parasites has evolved in response to cuckoo-host co-evolutionary interactions. Specifically, we predicted that cuckoos that exploit ejector hosts should display thicker eggshells relative to their hosts than cuckoos that exploit acceptor hosts, and thicker eggshells than predicted for their size^23–25^. Overall, we found that cuckoo eggshell thickness did not differ significantly either from host eggshell thickness or from the avian average for a given egg size. Similarly, when considering hosts with known egg ejection rates only, the eggshell thickness of cuckoos that exploit ejector hosts did not differ significantly from the eggshell thickness of cuckoos that exploit acceptor hosts. Previous research has shown that dome-nesting hosts tend to accept cuckoo eggs, whereas cup-nesting hosts tend to eject cuckoo eggs^37^. Therefore, we also investigated eggshell thickness in a larger sample of cuckoo hosts, for whom egg ejection rates were unknown but inferred based on nest type. Contrary to predictions, cuckoos that exploit dome-nesting hosts had significantly thicker eggshells relative to their hosts than cuckoos that exploit cup-nesting hosts.

In contrast to European and African cuckoos^24,31^, our data from Australian cuckoos does not support the hypothesis that thicker eggshells evolve in response to host egg ejection behaviour. A possible explanation for this is that ejector hosts may have adopted defence strategies that do not select for increased eggshell thickness in cuckoos. The host defence that is believed to select for thicker cuckoo eggshells is puncture ejection ^31^. However, there may be little selection for thicker cuckoo eggshells if hosts use grasp ejection (grasping the whole, undamaged egg in the mandible) rather than puncture ejection^38^. Grasp ejection is thought to be particularly constrained among hosts that have small mandibles^24^. While, the method of ejection is unknown for most Australian cuckoo hosts, it is interesting to note that the ejector hosts in our study were the larger-bodied species among the hosts and are therefore likely to be more capable of grasp-ejecting foreign eggs than the acceptor hosts.

Our finding that cuckoos that exploit dome-nesting hosts had significantly thicker eggshells relative to their hosts than cuckoos that exploit cup-nesting hosts is consistent with the hypothesis that thicker eggshells evolve in brood parasites to prevent breakage during egg laying. All acceptor hosts in this analysis build dome-shaped nests. Cuckoos that exploit these dome nesting hosts do not fully enter the nest, but leave the lower back, wingtips and tail outside the nest entrance during egg laying^39^, suggesting that the egg is dropped down from the nest entrance into the bowl of the nest. Thus, we might expect that these cuckoos would be under selection for thicker eggshells to avoid breakage during laying^23^.

Another possibility that remains to be tested, is whether embryonic behaviour can impact selective pressures on eggshells. For example, in European cuckoos, brood parasite embryos are stronger and exercise more whilst inside the egg^40^. Increased embryonic activity could conceivably result in selection for thicker eggshells in cuckoo species if thicker shells are associated with increased hatching success. However, nest architecture would need to be a strong predictor of embryonic activity to fully explain our observations that thicker relative eggshells only occur in dome-nesting species.

We did not find a significant difference in eggshell thickness relative to hosts between cuckoos that exploit acceptor and ejector hosts when considering hosts with known egg ejection rates only, although the non-significant trend was in the same direction as for the analysis considering hosts with both known and unknown egg ejection rates. This is likely to reflect insufficient power due to the small number of host species whose egg ejection rates have been quantified. However, this result is consistent with an earlier study that also found no difference in eggshell thickness of the *Cuculus/Cacomantis/Chalcites/Chrysococcyx* group of cuckoos and their hosts^26^. In Australian cuckoos, other factors could also be influential determinants of eggshell thickness, such as: diet, maternal age, habitat-dependent calcium availability^41^, chemical pollutants^42–45^ or the developmental environment^46^. Alternatively, egg strength may be enhanced through mechanisms other than thicker eggshells, such as denser shells^47^, or a stronger microstructure within the shell^48,49^. These possibilities remain to be tested for Australian cuckoo species.

We need to consider aspects of avian biology and our sampling strategy that may have impaired the power of our study to detect differences in eggshell thickness. Eggshell thickness is a physiologically constrained trait that must be maintained within the thresholds that allow embryo development. Thus, it is possible that there is insufficient variation in the phenotype for evolution to act effectively upon. However, we suggest that this is not the case due to the wealth of other studies that have demonstrated evidence for the evolution of eggshell thickness in avian and non-avian egg laying species^50–52^. Another point to consider is whether the co-evolutionary interactions between hosts and cuckoos in our study are sufficiently strong to select for changes to eggshell thickness. In the case of the bronze-cuckoos and their hosts in this analysis, the co-evolutionary relationship is very strong. The cuckoos specialise on their hosts and show highly specialised adaptations to their hosts, such as mimicry of host nestling skin colour and begging calls^9,13^. Similarly, the hosts show highly specialised adaptations to prevent cuckoo parasitism, such as a special alarm call that is only produced in the presence of cuckoos^9^, rejection of cuckoo chicks^5^ and early breeding to avoid parasitism^53^. The pallid cuckoo has been under sufficiently strong selection from egg rejection by hosts to have evolved several different races, each of which exploits a different host and lays an egg that mimics that of its preferred host^54^. Similarly, the koel has evolved eggs that closely mimic the appearance of those of one of its major hosts^55^. Thus, it seems likely that the co-evolutionary interactions between these cuckoos and their hosts have been sufficiently strong and long lasting to allow for selection on eggshell strength.

We studied eggshell thickness in more than a single host for all cuckoo-host comparisons. This means that the variation in eggshell thickness, even when normalised for egg size, is much larger among the host data than the cuckoo data. We could improve power by comparing eggshell thickness in only the most heavily exploited host species to the associated cuckoo species. This was unfortunately not possible in this study and would require nation-wide co-ordination of eggshell specimens to have a sufficient sample size for statistical comparisons. Additionally, if egg ejection rates were known for hosts of more Australian cuckoo species, this would improve power to test the puncture resistance hypothesis by comparing more than two cuckoo species for each type of host ejection behaviour (accept or eject). Ideally, investigating cuckoos that are phylogenetically distant to the current study species would improve confidence in our conclusions.

Our study is the first to apply Hall-effect magnetic-inference methodology to estimate eggshell thickness in museum eggshells without damage. We have shown that the non-destructive method is highly repeatable and provides direct and near continuous estimates of eggshell thickness at any point in the egg. This approach is an improvement upon previous methodologies that indirectly estimate eggshell thickness^56,57^ or use analog micrometers to measure thickness at a single location on the egg^58,59^. Importantly, Hall-effect magnetic-inference methodology is very transportable and allows measurements to be taken in the field or in situ at museums when specimens cannot be transported. It is less expensive and more accessible than scanning electron microscopy^60^ and micro computed tomography^61,62^, and complements other non-invasive approaches to estimate eggshell thickness in vivo^63,64^. Similar to previous benchmarking studies^65^, direct comparison of the precision and accuracy of all eggshell thickness estimation methods would be a valuable resource for the research community. By combining new digital technologies with the depth of historical collecting effort, our study has rapidly generated a large dataset suitable for comparative analyses (78 avian species; 34 families; 12 orders). Hall-effect magnetic-inference could facilitate researchers to take full advantage of the estimated 5 million egg specimens in collections world-wide^66^ and accelerate morphological, physiological, ecotoxicological, developmental and evolutionary research that relies upon accurate estimation of eggshell thickness traits.

Our results provide some support for the ‘impact resistance hypothesis’^23^, but further work is warranted. In particular, more data is needed on cuckoo-host biology, including rates of egg ejection and methods of egg ejection in the hosts of other species of cuckoos. Analysis of cuckoo eggshell structure and density may also be informative. Our study is likely to be underpowered due to inflated variation in host estimates and because we were restricted to studying only two cuckoo species for each type of host response to cuckoo eggs. Investigation of eggshell thickness restricting hosts to the most heavily exploited primary host and the addition of more Australian cuckoo-host pairs may add power to the trends observed here. This additional work will contribute to a greater understanding of the capacity for co-evolutionary pressures to drive phenotypic divergence.

## Methods

### Cuckoo and host species

The parasitic cuckoo species selected for use in this study were chosen based on previous knowledge of their host selection and the egg ejection behaviour of those hosts^15,37,67–71^. We selected four Australian cuckoo species based on the known egg ejection rates of their primary hosts from our earlier studies. Two congeneric species, Horsfield’s bronze-cuckoos *Chalcites basalis* and shining bronze-cuckoos *C. lucidus*, exploit hosts that build dome-shaped nests in which detection of foreign eggs is constrained by poor visibility in the dark interior^22,37^. Hosts of these two cuckoo species rarely eject either naturally-laid cuckoo eggs, or experimental, non-mimetic plastic model eggs, of similar size to their own (Table 1)^5,13,37^. The two other cuckoo species in the study, the pallid cuckoo (*Cuculus pallidus*) and the Pacific koel (*Eudynamis orientalis*), exploit hosts that build cup-shaped nests with good visibility, and these hosts routinely eject both naturally-laid cuckoo eggs and experimental, non-mimetic model eggs (Table 1)^37,72,73^.

**Table 1:** Characteristics of Australian cuckoo eggs and the eggs of their hosts used in this study. Summary statistics for morphological egg measurements, details of host species, and the rates of cuckoo egg ejection are reported for experimental, non-mimetic model eggs.

Cuckoo species	*N*	Mass (mg) ± SD	Length (mm) ± SD	Breadth (mm) ± SD	Apex thickness (um) ± SD	Meridian thickness (um) ± SD	Host species	*N*	Mass (mg) ± SD	Length (mm) ± SD	Breadth (mm) ± SD	Apex thickness (um) ± SD	Meridian thickness (um) ± SD	Nest type	Ejection behaviour	Ejection rate	References
*Chalcites basalis* (Horsfield’s Bronze-cuckoo)	3	79.33 ± 7.37	17.53 ± 0.19	12.00 ± 0.53	80.73 ± 5.27	86.18 ± 4.49	*Acanthiza chrysorrhoa* (Yellow-rumped thornbill)	2	71 ± 9.85	20.05 ± 5.92	14.57 ± 3.82	62.53 ± 1.70	79.18 ± 5.26	Dome/Closed	Acceptor	10%	Langmore et al.^37^, Medina & Langmore^4^
	2	83.5 ± 3.61	16.96 ± 1.16	11.86 ± 0.18	82.43 ± 3.42	98.04 ± 6.66	*Acanthiza reguloides* (Buff-rumped thornbill)	2	74 ± 0	17.25 ± 1.06	13.08 ± 0.24	78.03 ± 6.72	70.76 ± 3.31	Dome/Closed	Acceptor	0%	Langmore et al.^37^
	2	86.5 ± 2.12	17.54 ± 0.82	12.40 ± 0.54	89.54 ± 12.15	101.39 ± 10.90	*Aphelocephala leucopsis* (Southern whiteface)	2	94 ± 7.07	21.42 ± 3.90	14.95 ± 3.08	81.46 ± 0.43	101.52 ± 0.27	Dome/Closed	No Data	–	
	3	85.0 ± 3.61	24.44 ± 78.92	17.46 ± 5.89	80.62 ± 11.69	104.51 ± 15.29	*Acanthiza pusilla* (Brown thornbill)	1	75	16.19	11.67	77.81	95.85	Dome/Closed	Acceptor	12.50%	Langmore et al.^37^
	6	88.50 ± 5.89	18.67 ± 2.63	13.08 ± 2.56	88.16 ± 12.28	92.51 ± 6.95	*Malurus cyaneus* (Superb fairy-wren)	10	70.90 ± 4.79	19.32 ± 4.02	13.75 ± 2.89	78.71 ± 11.56	80.65 ± 6.97	Dome/Closed	Acceptor	10.60%	Langmore et al.^37^
	Mean	85.14 ± 3.40	19.09 ± 2.77	13.18 ± 2.14	81.92 ± 6.94	96.05 ± 6.63											
*Chalcites lucidus* (Shining bronze-cuckoo)	12	82.21 ± 7.35	19.33 ± 4.98	13.57 ± 3.23	79.33 ± 18.09	78.57 ± 9.48	*Acanthiza chrysorrhoa* (Yellow-rumped thornbill)	7	69 ± 7.79	20.45 ± 6.03	14.61 ± 3.66	64.88 ± 9.13	72.91 ± 5.73	Dome/Closed	Acceptor	10%	Langmore et al.^37^
	2	70.50 ± 0.71	17.29 ± 0.18	12.28 ± 0.41	65.07 ± 1.81	68.85 ± 3.24	*Acanthiza lineata* (Striated thornbill)	3	56.50 ± 4.36	25.24 ± 8.01	17.95 ± 5.51	61.65 ± 10.87	69.65 ± 4.40	Dome/Closed	No Data	–	
	7	86.21 ± 11.46	18.14 ± 0.71	12.66 ± 0.39	72.46 ± 8.66	78.36 ± 10.30	*Acanthiza pusilla* (Brown thornbill)	4	67.71 ± 5.79	23.40 ± 5.43	16.84 ± 3.81	61.06 ± 8.90	70.54 ± 7.10	Dome/Closed	Acceptor	12.50%	Langmore et al.^37^
	1	83	17.21	12.66	NA	81.7	*Malurus cyaneus* (Superb fairy-wren)	1	84	17.49	12.97	55.93	81.51	Dome/Closed	Acceptor	10.60%	Langmore et al.^37^
	Mean	80.45 ± 6.87	17.99 ± 0.99	12.79 ± 0.55	72.29 ± 7.13	76.87 ± 5.56											
*Heteroscenes pallidus* (Pallid cuckoo)	2	199.50 ± 12.02	23.20 ± 0.00	17.05 ± 0.47	100.55 ± 3.37	109.35 ± 5.61	*Artamus leucorynchus* (White-breasted woodswallow)	2	215.00 ± 4.95	17.10 ± 1.99	12.11 ± 2.12	106	113.58 ± 6.93	Cup/Open	No Data	–	
	3	238.33 ± 44.77	24.65 ± 0.51	17.62 ± 0.15	104.35 ± 27.80	115.69 ± 18.64	*Anthochaera carunculata* (Red wattlebird)	2	428.50 ± 30.41	28.12 ± 3.26	20.70 ± 2.84	150.09	144.76	Cup/Open	Ejector	42.90%	Langmore et al.^37^
	2	204.00 ± 24.04	24.17 ± 0.10	16.88 ± 0.45	89.02	103.03	*Gavicalis virescens* (Singing honeyeater)	1	139	30.93	21.23	81.92	100.73	Cup/Open	No Data	–	
	1	240	24.51	17.84	104.03	116.38	*Manorina flavigula* (Yellow-throated miner)	3	312.67 ± 81.77	20.46 ± 4.75	14.89 ± 3.34	148.30 ± 36.94	138.64 ± 4.85	Cup/Open	Ejector	*	Landstrom et al.^74^
	3	242.33 ± 7.51	24.23 ± 1.42	17.52 ± 0.30	88.40 ± 15.00	122.44 ± 6.89	*Melithreptus affinis* (Black-headed honeyeater)	2	105.50 ± 7.78	17.82 ± 0.33	13.02 ± 0.35	82.59 ± 9.76	93.81 ± 11.46	Cup/Open	Ejector	*	Starling et al.^54^
	2	221.00 ± 4.24	23.72 ± 0.14	16.99 ± 0.26	78.23 ± 3.20	112.32 ± 4.82	*Melithreptus lunatus* (White-naped honeyeater)	2	108.50 ± 20.51	17.77 ± 1.17	12.95 ± 1.25	76.36 ± 12.52	94.15 ± 10.41	Cup/Open	No Data	–	
	2	241.00 ± 35.36	24.45 ± 0.40	17.13 ± 1.43	91.84 ± 1.23	124.62 ± 4.29	*Melithreptus validirostris* (Strong-billed honeyeater)	2	170.00 ± 7.07	19.80 ± 2.26	15.27 ± 1.34	115.87 ± 23.35	115.78 ± 5.80	Cup/Open	No Data	–	
	2	231.00 ± 19.80	20.63 ± 5.08	8.77 ± 10.81	88.47 ± 4.94	114.72 ± 4.28	*Ptilotula fusca* (Fuscous honeyeater)	2	107.50 ± 6.36	27.17 ± 3.88	19.35 ± 1.84	70	81.40 ± 1.6	Cup/Open	No Data	–	
	3	244.00 ± 21.79	27.10 ± 4.79	19.68 ± 3.80	95.23 ± 14.67	119.92 ± 11.30	*Ptilotula ornata* (Yellow-plumed honeyeater)	1	104	16.8	11.87	NA	101.94	Cup/Open	No Data	–	
	1	179	22.51	17.03	87.39	NA	*Ptilotula plumula* (Grey-fronted honeyeater)	1	94	16.24	12.66	67.63	87.87	Cup/Open	No Data	–	
	1	239	24.02	18.52	107.29	116.28	*Microeca fascinans* (Jacky winter)	1	113	19.19	14.06	NA	103.7	Cup/Open	No Data	–	
	2	197.50 ± 30.41	22.72 ± 0.57	16.81 ± 0.49	90.65 ± 18.01	117.75 ± 7.02	*Rhipidura leucophrys* (Willie wagtail)	2	120.00 ± 18.38	24.55 ± 11.07	16.84 ± 6.15	93.00 ± 17.73	98.18 ± 11.65	Cup/Open	Ejector	36%	Landstrom et al.^72^
	Mean	223.06 ± 22.33	23.83 ± 1.54	16.82 ± 2.67	93.79 ± 8.65	115.68 ± 6.01											
*Eudynamys orientalis* (Eastern/Pacific koel)	6	621.43 ± 100.30	31.02 ± 6.15	22.16 ± 4.53	158.43 ± 12.76	165.85 ± 19.19	*Philemon citreogularis* (Little friarbird)	6	273.13 ± 25.24	27.14 ± 6.06	19.84 ± 4.44	126.19 ± 23.69	129.38 ± 8.99	Cup/Open	No Data	–	
	6	683.33 ± 35.12	31.20 ± 6.30	21.70 ± 4.61	158.89 ± 9.16	171.15 ± 11.97	*Philemon corniculatus* (Noisy friarbird)	5	429.80 ± 37.82	26.50 ± 7.83	18.89 ± 5.56	134.64 ± 15.57	146.50 ± 10.96	Cup/Open	Ejector	38% 4% 42.9%	Abernathy^55^ Abernathy et al.^73^ Langmore et al.^37^
	7	719.57 ± 55.21	32.29 ± 6.89	22.66 ± 4.73	163.31 ± 11.85	169.66 ± 6.52	*Grallina cyanoleuca* (Magpie-lark)	7	390.14 ± 34.13	23.47 + /0 5.79	17.02 ± 4.25	126.44 ± 6.66	144.43 ± 4.90	Cup/Open	Ejector	91% 89%	Abernathy^55^ Abernathy et al.^73^
	6	725.67 ± 78.74	34.56 ± 1.22	24.82 ± 1.07	152.55 ± 12.66	165.22 ± 6.52	*Sphecotheres vieilloti* (Australasian figbird)	7	512.86 ± 13.67	22.69 ± 7.05	16.57 ± 4.74	148.93 ± 21.46	154.02 ± 4.13	Cup/Open	No Data	–	
	Mean	687.5 ± 47.85	32.27 ± 1.63	22.84 ± 1.38	158.30 ± 4.42	167.97 ± 2.89											

*For these hosts, rates of egg ejection were unknown, but egg ejection behaviour by hosts could be inferred from quantitative analyses revealing that the cuckoo eggs are near perfect mimics of the host eggs, suggesting that the cuckoos have been subject to strong selection for egg mimicry through host egg ejection^54,72^.

In addition to their primary hosts included in this analysis, these cuckoos also exploit several secondary hosts whose egg ejection behaviour is unknown^67^. However, previous analyses indicate that there is a strong association between visibility inside the nest and egg ejection behaviour; hosts that build dome-shaped nests tend to accept foreign eggs (100% of Australian hosts [N = 6] ejected ≤ 25% of foreign eggs^37^), whereas hosts that build cup-shaped nests tend to eject foreign eggs (75% of hosts ejected > 25% of foreign eggs, [N = 8]^37^). Therefore, we conducted a second set of analyses that included both primary and secondary hosts of these cuckoos (Table 1), where egg ejection behaviour was inferred based on nest type for the secondary hosts.

### Eggshell measurements

All eggshells used in this study were sourced from the Commonwealth Scientific and Industrial Research Organisation (CSIRO) Australian National Wildlife Collection (ANWC) oology research collection (Supplementary data file 1). All eggs had been prepared at the time of collection by drilling a small hole in the shell, through which the egg contents were blown and removed. The eggshells were then washed and stored dry. Collector’s notes and consistently small blow-hole diameters indicate that eggs were sampled early in development and were unlikely to be subject to significant eggshell thinning during development (Supplementary data file 1). The availability of parasitised clutches in the ANWC collection dictated which host species were included and the sample size for this study (Table 1; Supplementary data file 1). Suitable cuckoo-host clutches contained at least one intact host and one cuckoo egg, both identified to the species level.

We used a precision industrial wall-thickness measuring tool to directly measure eggshell thickness via Hall-effect magnetic-inference, in a similar approach to Peterson et al.^75^. However, unlike this previous study we did not cut or damage the eggshell to take measurements. Specifically, we used the ElectroPhysik MiniTest FH7200 gauge and FH4 magnetic probe, with a 1.5 mm diameter steel ball which was inserted inside the empty eggshell, through the existing blow-hole in the specimen (SI 2.0). Thus, all eggs included in the study necessarily had a blowhole diameter > 1.5 mm. This probe and steel ball combination measures thicknesses up to 2 mm, with an accuracy of ± 3 µm + 1% of the reading (*Check Line*®, Germany). Thickness data was collected at a rate of 10 measurements per second. We did not place the steel probe in direct contact with the egg. Instead, we inserted a 0.73 mm sheet of plastic (cellulose acetate) in between the probe and the egg to minimise risk of damage (hereafter referred to as the ‘protector’).

Eggshell thickness data was collected at two regions on each egg—the apex (the most conical end opposite the air sac) and the meridian (the circumference around the widest part of the egg). Manual handling of the egg specimens during thickness estimation is described in detail in Supplementary Information Sect. 2.0 and Fig. S1. Briefly, we inserted the steel ball though the blow hole and rolled the ball to the apex of the egg. We always approached the magnetic probe (and protector) apex-first because this is the strongest part of the egg. Apex thickness was recorded for five seconds by leaving the egg stationary and untouched on the probe (Fig. S1; Video Supplement 1). We then rolled the ball until it was positioned adjacent to the side blow-hole and rotated the egg slowly, to record the meridian thickness (Fig. S1; Video Supplement 2). The steel ball was removed by rolling it back through the blow-hole, whilst still in contact with the probe (Video Supplement 3). Preliminary method optimisation using 60 unregistered eggs indicated that the risk of breaking an egg during this manual handling was very low if specimens had no pre-existing physical damage (cracks, chips, hairline fractures determined via illuminating the egg with a cold-light source) and weighed > 0.05 g (Fig. S2). No registered collection items sustained damage in this study.

All data were inspected and exported following the manufacturer’s protocols in the software package MSoft 7 Basic (*Check Line*®, Germany). The protector thickness was subtracted from the raw gauge readings to obtain a measurement of eggshell thickness (SI 2.0). Mean thickness (µm) was calculated for both apex and meridian measurements of each egg after removing outliers (classified as data points lying outside 1.5X the interquartile range). Mass (g), length (mm) and breadth (mm) were also measured for each egg. Length and breadth measurements were calculated from a 2D photograph of each egg, following Attard et al.^76^. Mass was measured using an electronic balance to the nearest 0.001 g (CT-250 On Balance Digital Scale).

### Repeatability Analysis

The repeatability of our Hall-effect magnetic-inference methodology with the ElectroPhysik probe was investigated by conducting replicate thickness measurements (*N* = 10) for an additional 10 unregistered eggs. Repeatability was calculated using the intra-class correlation coefficient (ICC), in the R package irr (SI 4.0). Significance was determined where *p* < 0.05.

### Comparative analysis of avian and cuckoo eggshell thickness

Previous studies indicate that eggshell thickness is positively correlated with egg size; larger eggs have thicker shells^77^. To investigate whether cuckoo eggshell thickness deviates from this general relationship, we calculated the mean eggshell thickness in a total of 78 species, comprising 12 avian orders and 34 families (total *N* = 3134 eggs) (Table 2). Our analysis included previously published data for 12 species^75^. We used a phylogenetic generalised least squares regression (PGLS) in the R package caper^78^, and estimated the relationship between eggshell length and two measures of thickness (apex and meridian). To control for phylogenetic relatedness, we used a maximum clade credibility (MCC) tree based on 100 trees downloaded from birdtree.org^79^. The MCC tree, which is the tree with the maximum product of the posterior clade probabilities, was obtained using the R package phangorn^80^. We extracted both phylogenetic residuals (phylogenetically independent) and residuals obtained from the phylogenetic regression line, and visually evaluated whether these residuals from cuckoo species were extreme values (e.g., were greater than expected by their size and phylogenetic position). We also used a linear regression of egg length versus mean eggshell thickness for 74 non-brood parasitic avian using the package lm in R v. 3.6.0^81^. Cuckoos were not included in this linear regression analysis and were plotted separately. We tested whether cuckoo values fell within the 95% confidence intervals of this regression.

**Table 2:** Egg size and eggshell thickness of bird species from 12 avian orders and 34 families.

Order	Family	Common name	Scientific name	Egg *N*	Length (mm)	Width (mm)	Meridian *N*	Mean meridian thickness (µM)	Apex *N*	Mean apex thickness (µM)	Data source
Accipitriformes	Accipitridae	Wedge-tailed eagle	*Aquila audax*	3	70.4	63.0	3	629.58	3	562.35	This study
Accipitriformes	Accipitridae	Little eagle	*Hieraaetus morphnoides*	3	55.2	42.8	3	409.39	3	373.12	This study
Accipitriformes	Pandionidae	Eastern osprey	*Pandion haliaetus*	2	62.6	45.9	2	641.17	2	665.69	This study
Anseriformes	Anatidae	Wood duck	*Aix sponsa*	39	49.9	38.0	39	326.00	38	120.00	Peterson et al.^75^
Anseriformes	Anatidae	Mallard	*Anas platyrhynchos*	2	56.7	40.9	2	339.00	2	337.00	Peterson et al.^75^
Caprimulgiformes	Apodidae	Australian swiftlet	*Aerodramus terraereginae*	1	21.7	13.0	1	103.75	1	115.86	This study
Charadriiformes	Burhinidae	Beach stone-curlew	*Esacus magnirostris*	1	62.0	41.3	1	397.66	0	NA	This study
Charadriiformes	Charadriidae	Western snowy plover	*Charadrius nivosus nivosus*	35	30.7	22.3	34	165.00	27	165.00	Peterson et al.^75^
Charadriiformes	Charadriidae	Red-kneed dotterel	*Erythrogonys cinctus*	1	29.7	23.0	1	184.34	0	NA	This study
Charadriiformes	Charadriidae	Inland dotterel	*Peltohyas australis*	1	37.9	26.7	1	235.66	1	201.11	This study
Charadriiformes	Laridae	Black noddy	*Anous minutus*	2	47.4	32.9	1	268.63	2	247.68	This study
Charadriiformes	Laridae	Caspian tern	*Hydroprogne caspia*	62	63.3	43.8	62	329.00	60	303.00	Peterson et al.^75^
Charadriiformes	Laridae	California gull	*Larus californicus*	175	65.0	45.3	175	352.00	162	348.00	Peterson et al.^75^
Charadriiformes	Laridae	Bridled tern	*Onychoprion anaethetus*	1	46.5	33.0	1	298.09	1	586.47	This study
Charadriiformes	Laridae	Black skimmer	*Rynchops niger*	11	47.8	34.6	11	247.00	11	226.00	Peterson et al.^75^
Charadriiformes	Laridae	Roseate tern	*Sterna dougallii*	2	40.5	28.1	2	246.85	2	404.55	This study
Charadriiformes	Laridae	Forster's tern	*Sterna forsteri*	1103	42.8	30.1	1085	203.00	946	194.00	Peterson et al.^75^
Charadriiformes	Laridae	California least tern	*Sternula antillarum browni*	340	30.7	22.4	332	144.00	249	140.00	Peterson et al.^75^
Charadriiformes	Recurvirostridae	Black-necked stilt	*Himantopus mexicanaus*	204	43.3	31.0	201	217.00	179	204.00	Peterson et al.^75^
Charadriiformes	Recurvirostridae	American avocet	*Recurvirostra americana*	844	49.2	34.1	843	242.00	773	226.00	Peterson et al.^75^
Columbiformes	Columbidae	Superb fruit-dove	*Ptilinopus superbus*	1	33.4	23.4	1	185.22	1	155.09	This study
Falconiformes	Falconidae	Brown falcon	*Falco berigora*	1	48.6	40.3	1	438.51	1	395.23	This study
Falconiformes	Falconidae	Nankeen kestrel	*Falco cenchroides*	1	39.4	28.2	1	290.03	1	249.01	This study
Galliformes	Phasianidae	Stubble quail	*Coturnix pectoralis*	1	31.2	22.5	1	286.75	0	NA	This study
Galliformes	Phasianidae	King quail	*Synoicus chinensis*	1	32.4	24.5	1	162.06	1	166.01	This study
Galliformes	Phasianidae	Brown quail	*Synoicus ypsilophorus*	1	29.8	24.5	1	325.67	0	NA	This study
Gruiformes	Rallidae	Lewin's rail	*Lewinia pectoralis*	2	35.6	26.8	2	207.31	2	174.61	This study
Passeriformes	Acanthizidae	Yellow-rumped thornbill	*Acanthiza chrysorrhoa *†	10	19.7	14.2	10	75.74	7	64.98	This study
Passeriformes	Acanthizidae	Striated thornbill	*Acanthiza lineata *†	2	26.2	18.7	2	66.01	1	69.91	This study
Passeriformes	Acanthizidae	Brown thornbill	*Acanthiza pusilla *†	7	22.9	16.6	6	69.18	5	64.406	This study
Passeriformes	Acanthizidae	Buff-rumped thornbill	*Acanthiza reguloides *†	2	17.3	13.1	2	70.76	2	78.03	This study
Passeriformes	Acanthizidae	Southern whiteface	*Aphelocephala leucopsis *†	2	21.4	15.0	2	101.52	1	81.77	This study
Passeriformes	Acanthizidae	Weebill	*Smicrornis brevirostris*	1	16.0	11.8	1	82.06	1	84.69	This study
Passeriformes	Artamidae	White-breasted woodswallow	*Artamus leucorynchus †*	2	18.5	13.6	2	118.48	0	NA	This study
Passeriformes	Campephagidae	Ground cuckoo-shrike	*Coracina maxima*	1	31.7	24.0	1	221.91	0	NA	This study
Passeriformes	Campephagidae	Black-faced cuckoo-shrike	*Coracina novaehollandiae*	1	32.5	23.8	1	160.94	1	178.38	This study
Passeriformes	Campephagidae	Cicadabird	*Edolisoma tenuirostre*	1	31.3	21.4	1	158.59	0	NA	This study
Passeriformes	Cisticolidae	Golden-headed cisticola	*Cisticola exilis*	1	15.8	11.4	1	90.85	0	NA	This study
Passeriformes	Climacteridae	Brown treecreeper	*Climacteris picumnus*	1	22.5	18.6	1	218.93	0	NA	This study
Passeriformes	Corcoracidae	White-winged chough	*Corcorax melanorhamphos*	1	42.5	28.0	1	235.24	1	255.66	This study
Passeriformes	Cracticidae	Australian magpie	*Gymnorhina tibicen*	1	41.1	28.2	1	214.08	1	201.65	This study
Passeriformes	Cuculidae	Horsfield’s bronze-cuckoo	Chalcites basalis*	18	19.2	13.4	18	95.27	18	83.94	This study
Passeriformes	Cuculidae	Shining bronze-cuckoo	Chalcites lucidus *	24	18.7	13.1	21	77.68	23	78.27	This study
Passeriformes	Cuculidae	Eastern/Pacific koel	Eudynamys orientalis*	27	32.2	22.8	22	168.30	26	158.50	This study
Passeriformes	Cuculidae	Pallid cuckoo	Heteroscenes pallidus*	24	24.0	16.8	20	116.48	23	94.22	This study
Passeriformes	Dicruridae	Spangled drongo	*Dicrurus bracteatus*	2	28.6	21.4	2	151.89	2	297.32	This study
Passeriformes	Maluridae	Superb fairy-wren	*Malurus cyaneus*†	13	19.2	13.7	12	80.73	11	76.43	This study
Passeriformes	Meliphagidae	Spiny-cheeked honeyeater	*Acanthagenys rufogularis*	1	25.4	17.2	1	129.86	1	110.88	This study
Passeriformes	Meliphagidae	Red wattlebird	*Anthochaera carunculata*†	2	28.1	20.7	2	144.76	1	150.09	This study
Passeriformes	Meliphagidae	Blue-faced honeyeater	*Entomyzon cyanotis*	1	32.0	22.0	1	178.90	1	155.86	This study
Passeriformes	Meliphagidae	White-fronted chat	*Epthianura albifrons*†	1	17.6	12.3	1	68.51	0	NA	This study
Passeriformes	Meliphagidae	Yellow-throated miner	*Manorina flavigula*†	3	22.7	16.9	4	137.85	1	121.49	This study
Passeriformes	Meliphagidae	Bell miner	*Manorina melanophrys*	1	22.4	16.5	1	119.29	1	96.84	This study
Passeriformes	Meliphagidae	Black-headed honeyeater	*Melithreptus affinis*†	2	17.8	13.0	2	93.81	2	82.59	This study
Passeriformes	Meliphagidae	White-naped honeyeater	*Melithreptus lunatus*†	2	17.8	13.0	2	94.16	2	76.36	This study
Passeriformes	Meliphagidae	Strong-billed honeyeater	*Melithreptus validirostris*†	2	19.8	15.3	2	115.78	2	115.87	This study
Passeriformes	Meliphagidae	Helmeted friarbird	*Philemon buceroides*	2	30.9	22.2	2	186.91	2	148.42	This study
Passeriformes	Meliphagidae	Little friarbird	*Philemon citreogularis*†	6	26.3	13.0	5	125.99	5	129.50	This study
Passeriformes	Meliphagidae	Noisy friarbird	*Philemon corniculatus*†	5	26.5	18.9	5	146.50	5	134.64	This study
Passeriformes	Meliphagidae	White-cheeked honeyeater	*Phylidonyris nigra*	1	19.8	15.0	1	96.57	1	93.05	This study
Passeriformes	Meliphagidae	Fuscous honeyeater	*Ptilotula fusca*†	2	27.2	19.4	2	81.40	0	NA	This study
Passeriformes	Meliphagidae	Yellow-plumed honeyeater	*Ptilotula ornata*†	1	16.8	11.9	1	101.94	0	NA	This study
Passeriformes	Meliphagidae	Grey-fronted honeyeater	*Ptilotula plumula*†	1	16.2	12.7	1	87.87	1	67.63	This study
Passeriformes	Monarchidae	Magpie-lark	*Grallina cyanoleuca*†	7	23.5	17.0	7	144.43	7	126.44	This study
Passeriformes	Oriolidae	Yellow oriole	*Oriolus flavocinctus*	1	31.7	22.7	1	168.40	1	172.91	This study
Passeriformes	Oriolidae	Australasian figbird	*Sphecotheres vieilloti*†	6	23.5	17.3	6	153.66	6	150.47	This study
Passeriformes	Pachycephalidae	Golden whistler	*Pachycephala pectoralis*	1	24.5	17.9	1	116.93	0	NA	This study
Passeriformes	Petroicidae	Eastern yellow robin	*Eopsaltria australis*	1	21.5	16.4	1	104.90	1	75.60	This study
Passeriformes	Petroicidae	Grey-headed robin	*Heteromyias cinereifrons*	2	26.0	18.6	2	118.63	2	132.61	This study
Passeriformes	Petroicidae	Jacky winter	*Microeca fascinans*†	1	19.2	14.1	1	103.70	0	NA	This study
Passeriformes	Petroicidae	Red-capped robin	*Petroica goodenovii*	1	14.6	12.4	0	NA	1	74.23	This study
Passeriformes	Pomatostomidae	Chestnut-crowned babbler	*Pomatostomus ruficeps*	1	26.0	18.5	1	171.45	0	NA	This study
Passeriformes	Ptilonorhynchidae	Spotted bowerbird	*Chlamydera maculata*	2	27.4	19.7	1	216.63	2	157.81	This study
Passeriformes	Rhipiduridae	Willie wagtail	*Rhipidura leucophrys*†	2	24.6	16.8	2	98.18	2	93.00	This study
Passeriformes	Turdidae	Eurasian blackbird	*Turdus merula*	1	29.5	21.0	1	194.97	1	224.69	This study
Pelecaniformes	Ardeidae	Great egret	*Ardea alba*	3	59.7	40.5	3	296.00	3	303.00	Peterson et al.^75^
Psittaciformes	Psittaculidae	Scaly-breasted lorikeet	*Trichoglossus chlorolepidotus*	1	25.8	20.9	1	184.32	1	151.62	This study
Suliformes	Phalacrocoracidae	Double-crested cormorant	*Phalacrocorax auritus albociliatus*	90	61.0	39.0	88	418.00	89	394.00	Peterson et al.^75^

*Brood parasitic cuckoo species.

^†^Host species of the cuckoos in this study.

### Statistical analysis

The distribution of raw and normalised eggshell thickness in cuckoos and their hosts was plotted and visually inspected in ggplot2 (Figs. S3 and Fig. 2). Within a species, outliers in the distribution of mean thicknesses (as defined as above) were removed. To account for inter-specific differences in egg size (which is correlated with eggshell thickness) ‘normalised thickness’ was calculated for each sample by dividing the eggshell thickness by egg length^75^. This approach is expected to successfully normalise the data because egg length explains a large proportion of the inter-species variation in eggshell thickness (Fig. 3). We tested for successful normalisation by regressing normalised eggshell thickness against eggshell mass (Fig. S4).

We tested whether, overall, the thickness of cuckoo eggshells relative to their length differed from that of their hosts at both the apex and the meridian of the egg using a Wilcoxon signed-rank test on matched pairs of cuckoo and host eggs. For this analysis, any unpaired egg samples, or samples with data missing for either the host or cuckoo of the pair were removed from analysis. Final sample sizes for each treatment can be found in Fig. 1. We then tested whether host ejection behaviour predicted the ratio of cuckoo to host normalised eggshell thickness. We used a Restricted Maximum Likelihood Model (REML), with cuckoo:host normalised eggshell thickness ratio as the response variable, host response to foreign eggs (accept or eject) as the fixed effect and host species nested within cuckoo species as the random effect. For all models we checked standardised residuals for normality. Log_10_ transformation of variables improved the normality of residuals (Anderson Darling Tests for Goodness-of-fit, all *P* > 0.4), so we present these results, although the qualitative results remained unchanged regardless of whether or not data were transformed. We ran four models; (1) eggshell thickness at the meridian including only hosts with known egg ejection rates, (2) eggshell thickness at the meridian including hosts with both known and unknown egg ejection rates, (3) eggshell thickness at the apex including only hosts with known egg ejection rates, and (4) eggshell thickness at the apex including hosts with both known and unknown egg ejection rates. The analyses were run in JMP v.15 (SAS Institute Inc, 2019).

## Supplementary Information

Supplementary Information 1.

Supplementary Information 2.

Supplementary Information 3.

Supplementary Information 4.

Supplementary Information 5.

Supplementary Information 6.

## Data Availability

Raw data can be accessed through DataDryad. Correspondence and requests for material should be addressed to CEH (clare.holleley@csiro.au) or NEL (naomi.langmore@anu.edu.au).
