# Supplementary material for: Thicker eggshells are not predicted by host egg ejection behaviour in four species of Australian cuckoo
Source: Sci Rep. 2022 Apr 15;12:6320. doi: 10.1038/s41598-022-09872-9 (PMC9012832; doi:10.1038/s41598-022-09872-9)
Supplement: Supplementary file 1 — Supplementary Information 1. [file 41598_2022_9872_MOESM1_ESM.pdf]

Supplementary Information

Thicker eggshells are not predicted by host egg ejection behaviour in four species of Australian cuckoo

Clare E. Holleley[1*], Alice C. Grieve[1], Alicia Grealy[1,2], Iliana Medina[3], Naomi E. Langmore[2*]

[1] Australian National Wildlife Collection, National Research Collections Australia, CSIRO, Canberra, ACT 2601, Australia
[2] Langmore Group, Research School of Biology, Building 46, Australian National University, Canberra, ACT 0200, Australia
[3] School of BioSciences, University of Melbourne, Royal Parade, VIC 3010, Australia

* Corresponding author. E-mail: clare.holleley@csiro.au and naomi.langmore@anu.edu.au

1.0 Samples used

All samples and associated ANWC registration numbers are listed in supplementary data file 1. Raw data from the Electrophysik probe is archived on DRYAD.

2.0 Measuring eggshell thickness

2.1 Gauge set up

(1) Lay felt on work surface
(2) Set up probe stand and plug probe into base unit
(3) Plug base unit into windows laptop and open MSoft 7
    Start Menu > All Programs > MSoft 7 > MSoft 7
(4) Turn on base unit & ensure **loudspeaker** is OFF
    Main Menu > Setup > User Preferences > Loudspeaker > *OK* > "OFF" > *OK*
(5) Check **date & time** has correct day & hour values
    Main Menu > Setup > Date / Time
(6) Check **data logging** settings are correct
    Main Menu > Active Batch > Configuration
        Target Ball Size: 1,5 mm
        Data logging mode: Auto
        Logging Rate: 10 / sec
(7) Check **calibration** is correct
    Main Menu > Active Batch > Calibration
        Target Ball Size: 1,5 mm
        Calibration Mode: Zero + 3Points
(8) All 4 calibration points should have an "X" in the box to the right under 'CAL', if they do not the probe needs to be re-calibrated:
    Zero: zero calibration cap for 1.5mm ball
        Calibration Mode > Zero > *OK*
        Place 1.5mm ball in cap
        Place cap (with ball) onto probe, let go and wait until a reading appears
        Lift cap (with ball) at least 3cm from probe, then place back onto probe
        Repeat until *n* = 5
        Press *OK* to save calibration

Point 1: calibration cap 278,3 µm ± 0,5%

Calibration Mode > Calibr. Point 1 > *OK*
Place 1.5mm ball in cap
Place cap (with ball) onto probe, let go and wait until a reading appears
Lift cap (with ball) at least 3cm from probe, then place back onto probe
Repeat until $n = 5$
Press *OK* to save calibration

Point 2: calibration cap 1,033 µm ± 0,5%

Calibration Mode > Calibr. Point 2 > *OK*
Place 1.5mm ball in caps
Place cap (with ball) onto probe, let go and wait until a reading appears
Lift cap (with ball) at least 3cm from probe, then place back onto probe
Repeat until $n = 5$
Press *OK* to save calibration

Point 3: calibration cap 3,00 µm ± 0,5%

Calibration Mode > Calibr. Point 3 > *OK*
Place 1.5mm ball in caps
Place cap (with ball) onto probe, let go and wait until a reading appears
Lift cap (with ball) at least 3cm from probe, then place back onto probe
Repeat until $n = 5$
Press *OK* to save calibration

All calibration points will be calculated from mean of 5 measurements, e.g.:

	1	2	3	4	5	$\bar{x}$	CAL
ZERO (µm)	-1	-0.9	-0.9	-1.5	-1.8	0	X
POINT 1 (µm)	269	268.5	270	269.5	269.5	269.5	X
POINT 2 (µm)	1.018	1.014	1.016	1.016	1.018	1.016	X
POINT 3 (µm)	3.04	3.01	3	3.01	3.01	3.02	X

(9) Place cradle and plastic protector over probe and tighten screw until the cradle is stable and the protector is taut over the probe point
(10) Measure and record the protector thickness

2.2 Data collection

(1) In MSoft 7, double-click MiniTest USB Adapter (130886) under Gauge Explorer to activate data logging
(2) Double-click DirectMode under MiniTest 7200FH
(3) Navigate to the correct file location in the explorer window
(4) Click Direct Import from the top menu

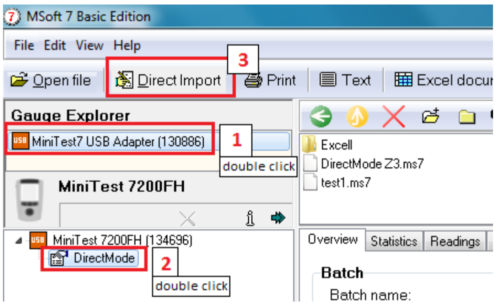

(5) Direct Import window will open

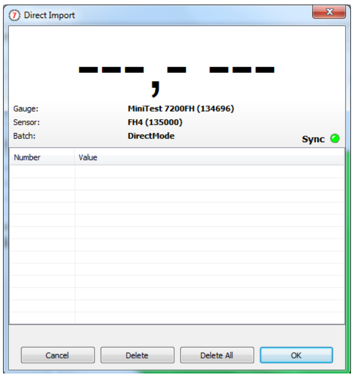

(6) Press *OK* on the **base unit** to begin data logging, press *OK* on the **base unit** to stop
N.B. if you click OK on the screen instead of the base unit to stop the data logging MSoft 7 will freeze and the data will be lost
If looking for specific *n* value, watch the number column in the direct import window until it reaches the necessary *n* value

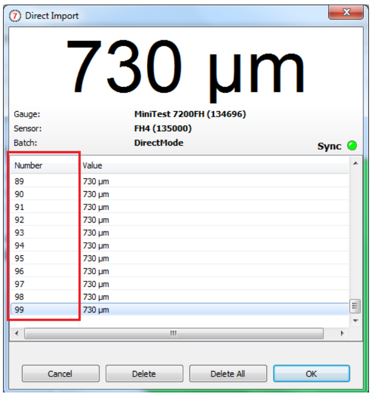

(7) When ready to save readings, click *OK* on direct import window

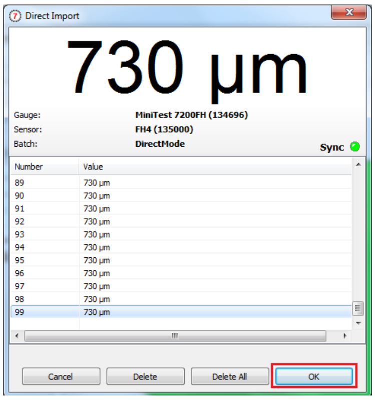

(8) Navigate to the correct file location, rename the file based on the specimen number and measurement, then confirm the file is saving in the correct format (.ms7) (e.g. E12345_APEX)

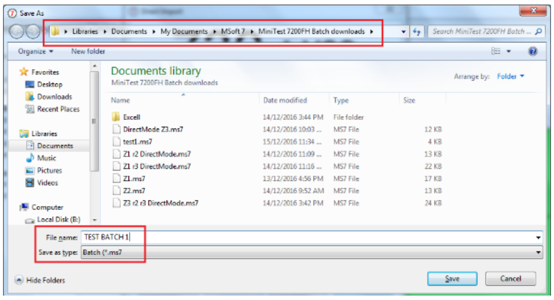

(9) The direct import window will close, and the new file will be visible under the File Explorer window, clicking that file will activate and display it in the Preview window (where Statistics, Histogram & Trend diagram can be viewed)

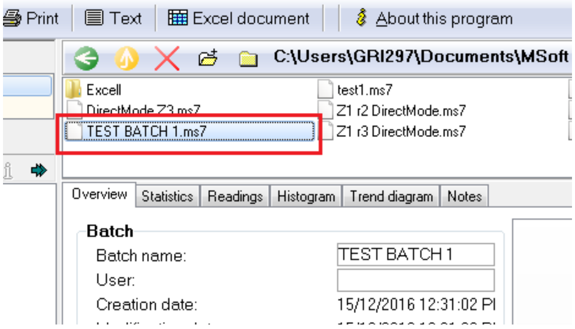

(10)To take another batch of readings simply click Direct Import again to reopen the window and repeat the process – it is wise to log all the batch readings for each specimen before exporting the files to Excel in groups

(11)To export readings to Excel (necessary to correct calculations for protector thickness) click Excel document while the active batch is selected

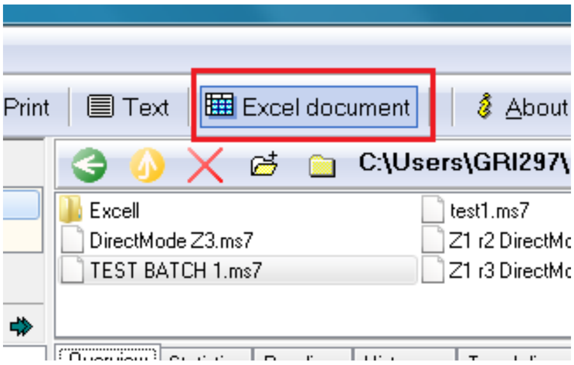

(12)Navigate to the correct file location, confirm the file is correctly named based on the specimen number and measurement being taken and change the file extension to the correct format (**.xls NOT .xlsx or .csv**)

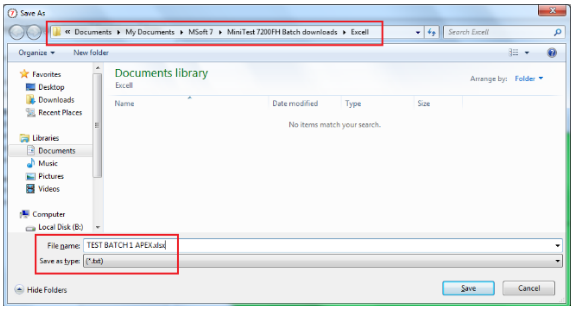

(13) Excel spreadsheet will automatically open (but this can be ignored for now)
(14) Always copy & save MSoft 7 and Excel files from the laptop to a USB

2.3 Manual handling

(1) Check whether blowhole is large enough to fit 1.5 mm ball without damage, and for jagged edges as a sign of weakness/propensity to crack
(2) Candle eggs
    Check which end is the base and which is the apex – the base will be apparent by the appearance of a darker circle where the membrane air pocket was located
    Check for fractures and membrane issues – any hairline fractures or thin patches will show as lighter and membrane or other biological material will show as darker
(3) Note: If the blowhole is too small, jagged, or there are visible issues when candling, the egg is at risk of damage and should not be measured
(4) Weigh the egg
(5) Photograph the egg
(6) Gently place 1.5 mm ball into egg through blowhole
(7) Gently bring the egg to the probe point with the conical end of the egg pointing down
(8) Leave egg to balance (do not touch while measuring) with the most conical point touching the probe
(9) Take roughly $n = 50$
(10) Gently move egg over probe point until ball is adjacent to the blowhole at the centre line of the egg, with the blowhole on the far side of the probe point from the person measuring. Always hold the egg with the conical apex end to the right of the person measuring. Slowly and smoothly rotate the egg, moving the blowhole away from the person measuring (in an anti-clockwise direction) until the blowhole is adjacent to the measurer on the near side of the probe – it is best to use a hand-over-hand technique when rotating to prevent jerky movements of the egg. Note that the time (and n value) will vary with the size of the egg for this measurement

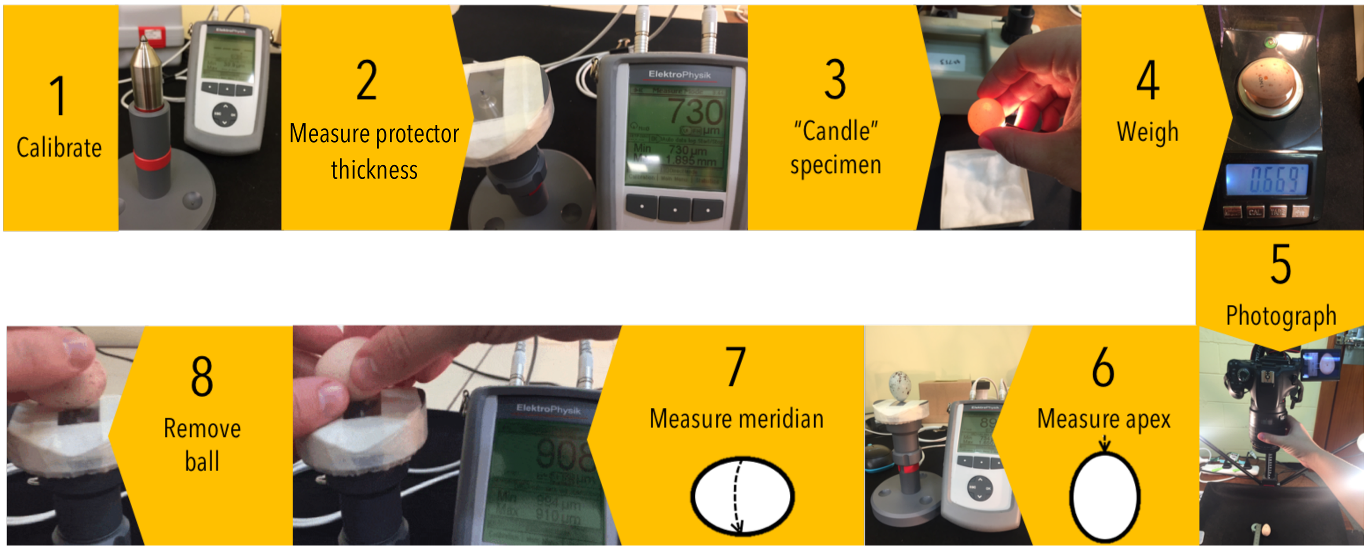

Figure S1. Steps in measuring the thickness of museum eggshell specimens. Thickness was directly estimated using Hall-effect magnetic-inference with an ElectroPhysik MiniTest FH7200 gauge, an FH4 magnetic probe and a 1.5 mm diameter steel ball at a rate of 10 measurements per second and an accuracy of ± 3 µm + 1% of the reading.

2.4 Data processing

(1) Open the .xls file for the batch of interest in Excel and navigate to the ‘Readings (Total)’ sheet
(2) Insert a new column between Reading and Unit (right-click column C header > Insert)

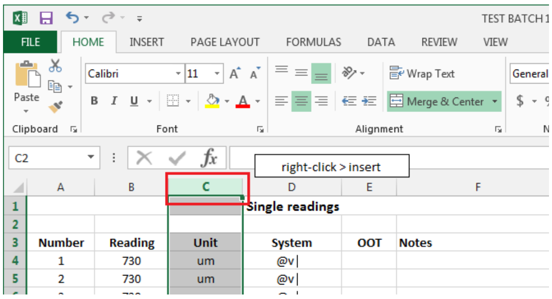

(3) Name the new column “corrected for protector”
(4) Enter equation to subtract mean protector calibration previously calculated from values in Reading column
(5) Place mouse over bottom-left corner of cell until the cursor turns into a solid black cross (+), click and drag down to last row of the Reading column
(6) Use the functions in Excel to calculate the following statistics:

Mean $\bar{x}$ (μm)	=AVERAGE(C4:CX)
Standard deviation s (μm)	=STDEV(C4:CX)
Median (μm)	=MEDIAN(C4:CX)
Max (μm)	=MAX('Readings (Total)'!C4:CX)
Min (μm)	=MIN('Readings (Total)'!C4:CX)
Range (μm)	=B6-B7
Q1 (Quartile 1)	=QUARTILE(C4:CX,1)
Q3 (Quartile 3)	=QUARTILE(C4:CX,3)
IQR (Inter-quartile range)	Quartile 3 - Quartile 1
Lower	Mean – (1.5*IQR)
Upper	Mean + (1.5*IQR)

CX = the last row of the Reading column – this will vary with the number of data points in the batch (*n*)
The equations in column 2 of the table above can be copies & pasted into Excel, but will all show as “#NAME?” until CX is changed to an actual cell value
The coefficient of variation is calculated as a percentage using the following equation:

$$V = \left({}^{s}/_{\bar{x}}\right) * 100\%$$

3.0 Damage incurred to eggshell specimens

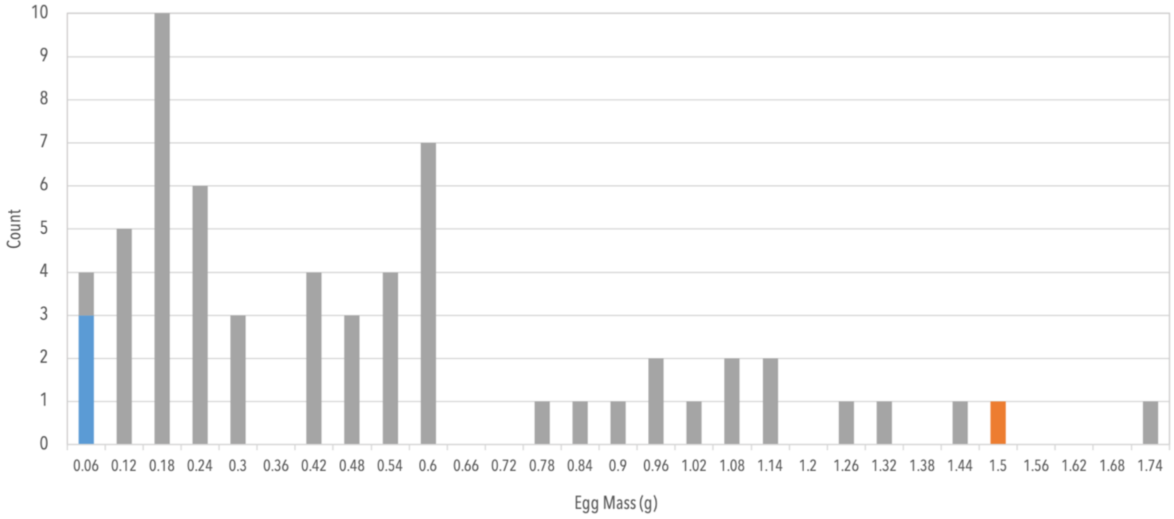

Figure S2. Frequency histogram showing egg breakage occurrence during magnetic thickness measurement. Most eggs were not damaged by manual handling and measurement (grey bars; 57 of 61 eggs undamaged; 93.4%). Three breakages were observed in eggs weighing < 0.06 g which had no pre-existing damage (blue bar). The only breakage that occurred in a relatively large egg was a single specimen with a pre-existing hair-line fracture (orange bar).

4.0 R code for repeatability analysis

Please see the DataDryad package for R inputs.

4.1 Apex

```
#Import data
> dat3<-read.csv("Apex_repeatability.csv",header=TRUE)

#Display data header
> head(dat3)
      X1     X2     X3     X4     X5     X6     X7     X8     X9    X10
1  59.85  74.61  97.28  79.20  68.00  62.60  68.35  64.15  78.12  65.82
2 173.09 171.96 171.31 131.93 108.13 110.72 115.48 141.44 129.54  95.33
3 140.29 123.18 144.94 124.04 144.74 134.79 132.31 119.84 134.76 140.52
4  86.53  91.06 102.24  94.64  79.79  83.77  80.82  98.37 115.86  98.38
5 103.00 104.12 102.24 146.76 122.03  99.52 112.28 100.84 114.63 116.44
6 102.42 105.24 105.50 113.97  96.44 110.84  86.32  94.64 107.49  88.56

#Load R library irr
> library(irr)

#Run inter-class correlation analysis
> icc(dat3,model="oneway",type="consistency", unit="average")
 Average Score Intraclass Correlation

   Model: oneway
   Type : consistency

   Subjects = 10
     Raters = 10
    ICC(10) = 0.961

 F-Test, H0: r0 = 0 ; H1: r0 > 0
    F(9,90) = 25.6 , p = 3.22e-21

 95%-Confidence Interval for ICC Population Values:
  0.912 < ICC < 0.989
```

4.2 Meridian

```
#Import data
>dat4<-read.csv("Meridian_repeatability.csv",header=TRUE)

#Display data header
> head(dat4)

      X1     X2     X3     X4     X5     X6     X7     X8     X9    X10
1  77.63  79.48  76.88  70.45  76.55  76.10  80.99  78.86  74.03  69.54
2  94.78  80.24 146.41  74.50  69.18  71.26  62.92  62.37  68.70  66.29
3  83.76  86.92  83.85  88.45  89.13  89.37  89.00  99.93  78.20  78.06
4  96.56  99.50  99.72  96.81  91.97  94.81  92.58  90.51 104.45  92.29
5 113.70 113.43 112.62 116.89 113.91 112.45 110.21 113.86 119.08 108.89
6 113.79 114.64 125.71 115.53 114.74 111.29 110.38 108.32 126.04 106.81

#Load R library irr
> library(irr)

#Run inter-class correlation analysis
> icc(dat4,model="oneway",type="consistency", unit="average")
 Average Score Intraclass Correlation

   Model: oneway
   Type : consistency

   Subjects = 10
     Raters = 10
    ICC(10) = 0.961

 F-Test, H0: r0 = 0 ; H1: r0 > 0
    F(9,90) = 25.9 , p = 2.2e-21

 95%-Confidence Interval for ICC Population Values:
  0.913 < ICC < 0.989
```

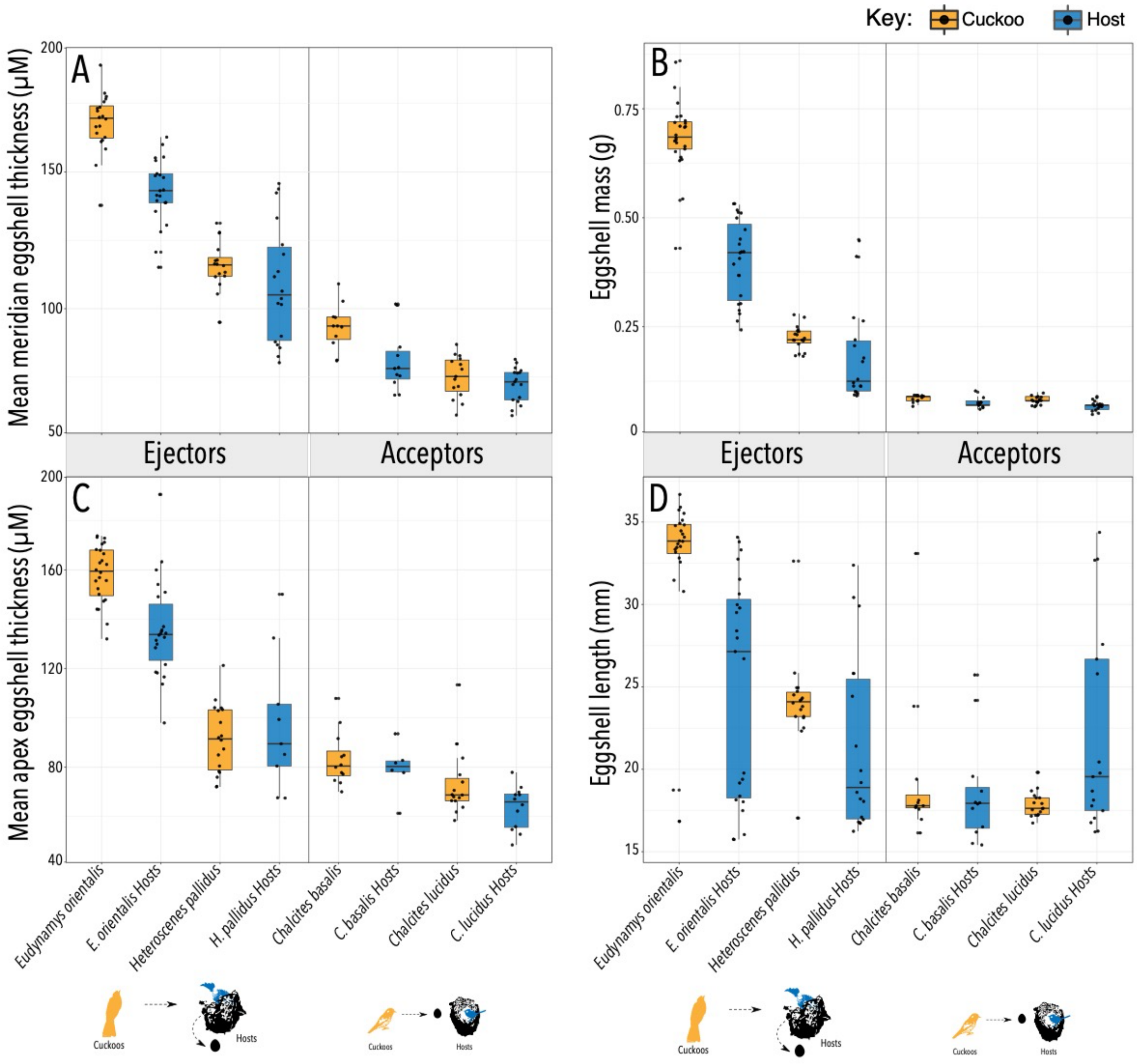

Figure S3. Eggshell morphology of cuckoos (orange) and their hosts (blue). Eggshell thickness was measured at two points on the egg: (A) Eggshell thickness at the meridian of the egg, which is the circumference around the widest part of the egg. (B) Eggshell mass. (C) Eggshell thickness at the apex of the egg, which is the most conical end opposite the air sac. (D) Eggshell length from apex to the blunt end of the egg. The total distribution is displayed untransformed as block dots. Box plots are the median, interquartile range (Q1 - Q3) and range (min – max).

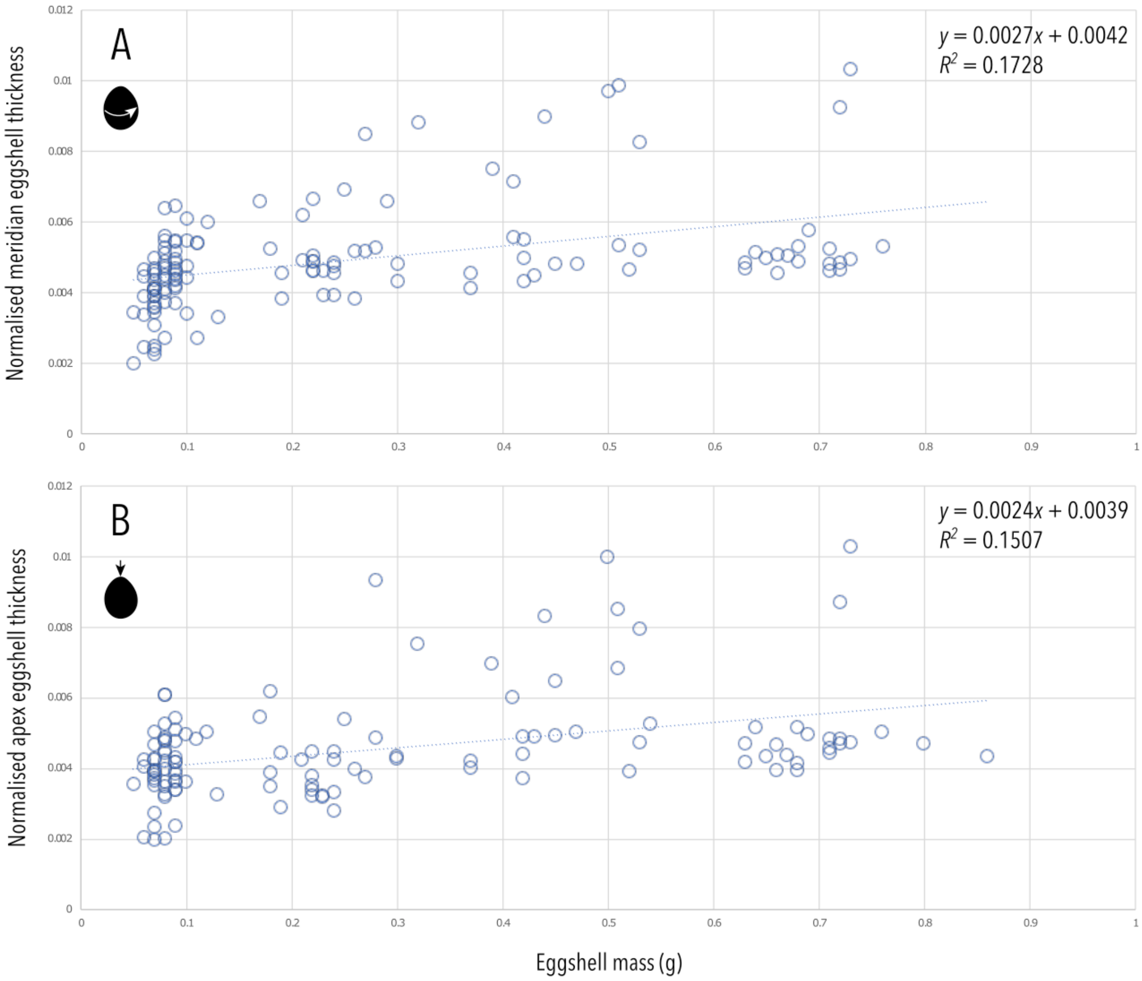

Figure S4. Normalisation of eggshell thickness measurements. Egg size (using mass as a proxy), is not a strong predictor of normalised eggshell thickness. For both (A) meridian and (B) apex measurements, the slope approaches zero (0.0024 – 0.0027) and the R^2 is low (0.15 – 0.17). This indicates that raw eggshell thickness was successfully normalised by dividing eggshell thickness by egg length.

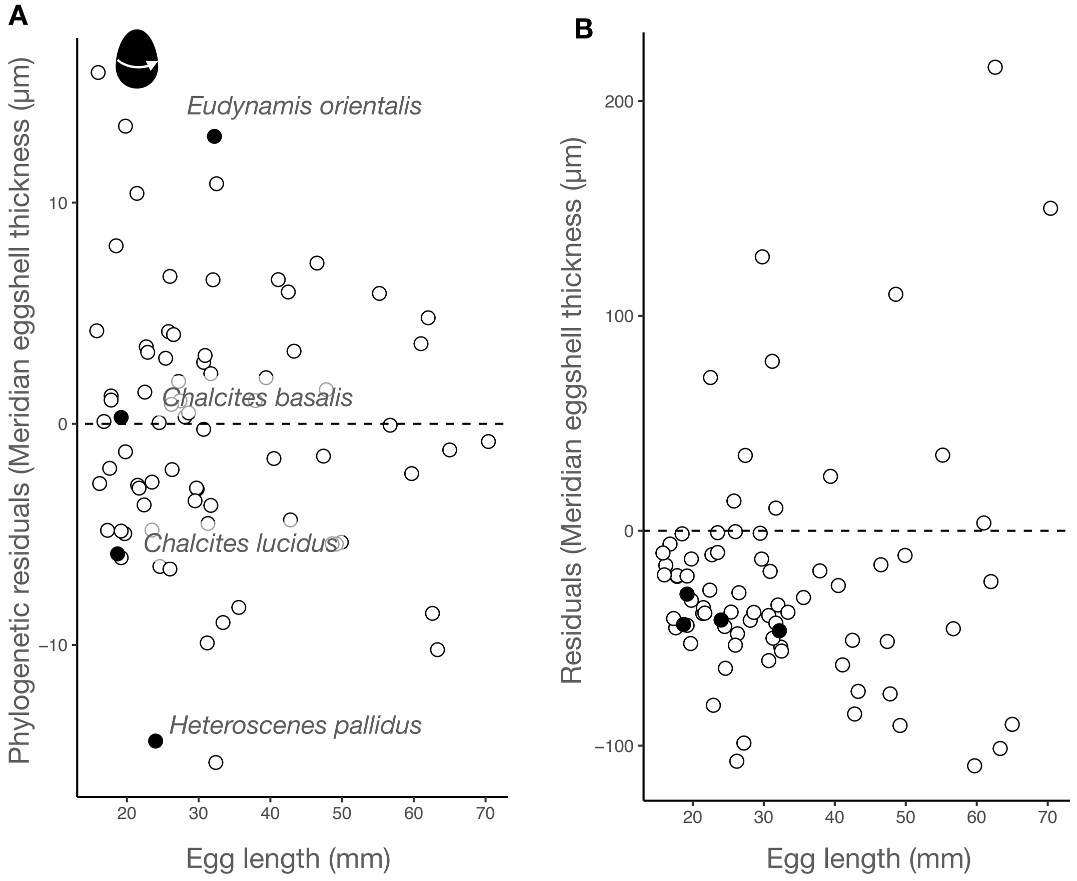

Figure S5. Distribution of residuals of meridian thickness and size relationship. (A) Phylogenetic residuals from PGLS on egg length and meridian eggshell thickness. Each point is phylogenetically independent. (B) Residuals estimated from regression equation in PGLS model, where the slope and intercept have been adjusted to control for phylogenetic relationships in dataset (*N* = 63).

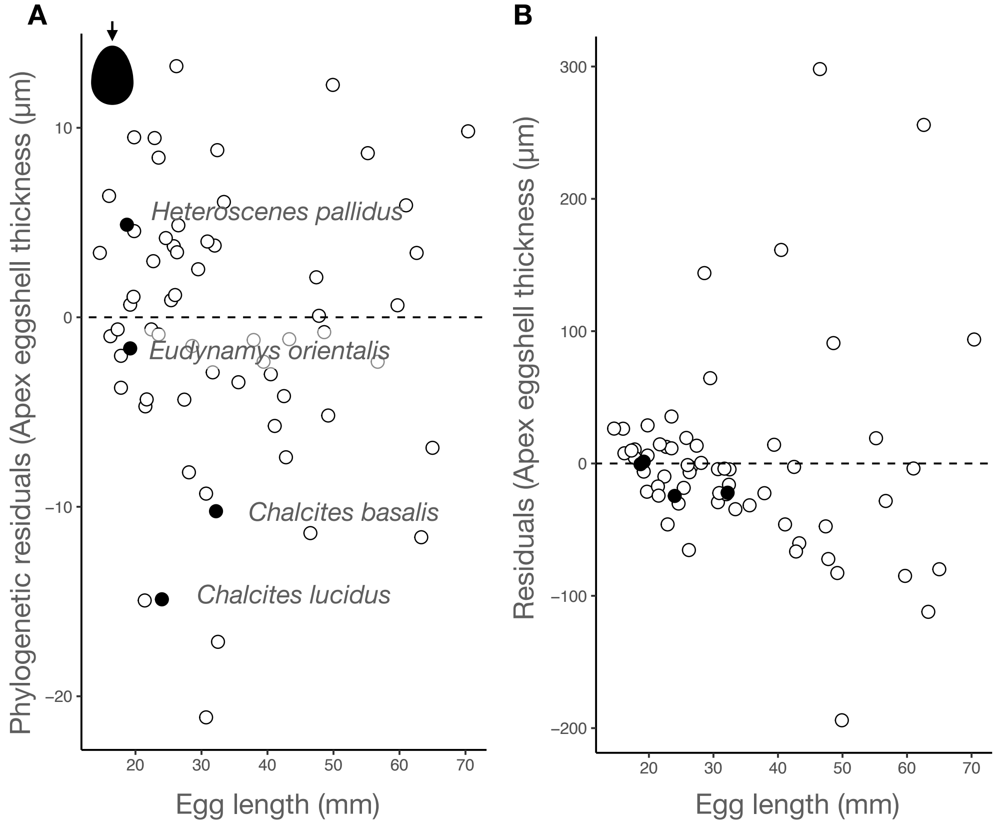

Figure S6. Distribution of residuals of apex thickness and size relationship. (A) Phylogenetic residuals from PGLS on egg length and apex eggshell thickness. Each point is phylogenetically independent. (B) Residuals estimated from regression equation in PGLS model, where the slope and intercept have been adjusted to control for phylogenetic relationships in dataset ($N = 77$).
