# Supplementary material for: Thicker eggshells are not predicted by host egg ejection behaviour in four species of Australian cuckoo
Source: Sci Rep. 2022 Apr 15;12:6320. doi: 10.1038/s41598-022-09872-9 (PMC9012832; doi:10.1038/s41598-022-09872-9)
Supplement: Supplementary file 2 — Supplementary Information 2. [file 41598_2022_9872_MOESM2_ESM.docx]

**Supplementary information titles and legends**

**Supplementary data file 1.** Data file containing information on all know hosts and egg ejection data, museum eggshell registration numbers (CSIRO Australian National Wildlife Collection) and associated metadata, raw morphological eggshell measurements and the input files used for REML analysis.

**Figure S1. Steps in measuring the thickness of museum eggshell specimens.** Thickness was directly estimated using Hall-effect magnetic-inference with an ElectroPhysik MiniTest FH7200 gauge, an FH4 magnetic probe and a 1.5 mm diameter steel ball at a rate of 10 measurements per second and an accuracy of ± 3 µm + 1% of the reading.

**Figure S2. Frequency histogram showing egg breakage occurrence during magnetic thickness measurement.** Most eggs were not damaged by manual handling and measurement (grey bars; 57 of 61 eggs undamaged; 93.4%). Three breakages were observed in eggs weighing < 0.06 g which had no pre-existing damage (blue bar). The only breakage that occurred in a relatively large egg was a single specimen with a pre-existing hair-line fracture (orange bar).

**Figure S3. Eggshell morphology of cuckoos (orange) and their hosts (blue)**. Eggshell thickness was measured at two points on the egg: (A) Eggshell thickness at the meridian of the egg, which is the circumference around the widest part of the egg. (B) Eggshell mass. (C) Eggshell thickness at the apex of the egg, which is the most conical end opposite the air sac. (D) Eggshell length from apex to the blunt end of the egg. The total distribution is displayed untransformed as block dots. Box plots are the mean and 95% confidence interval.

**Figure S4. Normalisation of eggshell thickness measurements.** Egg size (using mass as a proxy), is not a strong predictor of normalised eggshell thickness. For both (A) meridian and (B) apex measurements, the slope approaches zero (0.0024 – 0.0027) and the *R^2^* is low (0.15 – 0.17). This indicates that raw eggshell thickness was successfully normalised by dividing eggshell thickness by egg length.

**Figure S5. Distribution of residuals of meridian thickness and size relationship.** (A) Phylogenetic residuals from PGLS on egg length and meridian eggshell thickness. Each point is phylogenetically independent. (B) Residuals estimated from regression equation in PGLS model, where the slope and intercept have been adjusted to control for phylogenetic relationships in dataset (*N* = 63).

**Figure S6. Distribution of residuals of apex thickness and size relationship.** (A) Phylogenetic residuals from PGLS on egg length and apex eggshell thickness. Each point is phylogenetically independent. (B) Residuals estimated from regression equation in PGLS model, where the slope and intercept have been adjusted to control for phylogenetic relationships in dataset (*N* = 77).

**Video Supplement 1. Apex eggshell thickness measurement.** Apex thickness is measured by leaving the egg vertical and stationary on the probe for five seconds. Then the egg is slowly rotated to position the steel ball for the meridian measurement.

**Video Supplement 2. Meridian eggshell thickness measurement.** Measurements commence when the steel ball is positioned adjacent to the side blow-hole and are measured continuously as the egg is rotated slowly 360˚ around the widest part of the egg.

**Video Supplement 3. Removing the steel ball from the interior of the eggshell.** The steel ball is removed by rolling it back through the blow-hole, after completing a 360˚ meridian thickness measurement. The steel ball remains in contact with the probe at all times.
