# Supplementary figures and images for: Thicker eggshells are not predicted by host egg ejection behaviour in four species of Australian cuckoo
Source: Sci Rep. 2022 Apr 15;12:6320. doi: 10.1038/s41598-022-09872-9 (PMC9012832; doi:10.1038/s41598-022-09872-9)

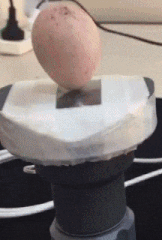

Supplement: Supplementary file 4 — Supplementary Information 4. [file 41598_2022_9872_MOESM4_ESM.gif]

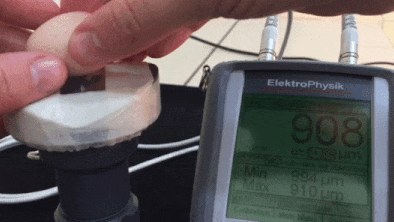

Supplement: Supplementary file 5 — Supplementary Information 5. [file 41598_2022_9872_MOESM5_ESM.gif]

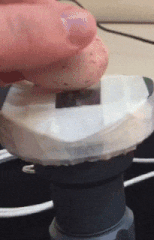

Supplement: Supplementary file 6 — Supplementary Information 6. [file 41598_2022_9872_MOESM6_ESM.gif]
